# Assessment of Serum Cystatin C Levels in Newly Diagnosed Acute Myocardial Infarction at the Onset and at the Time of Hospital Discharge

**DOI:** 10.14740/cr377w

**Published:** 2015-02-09

**Authors:** Adil H. Alhusseiny, Marwan S. M. Al-Nimer, Sarah Isam Attallah Al-Neamy

**Affiliations:** a Department of Medicine, College of Medicine, Diyala University, Diyala, Iraq; b Department of Pharmacology, College of Medicine, Al-Mustansiriya University, Baghdad, Iraq; c Janen Hospital, Baghdad, Iraq

**Keywords:** Acute myocardial infarction, Cystatin C, Cardiometabolic risk factors

## Abstract

**Background:**

Cystatin C (Cys-C) is a marker of renal damage. Higher serum levels of Cys-C were observed in cardiovascular disease. This study aimed to test the null hypothesis that Cys-C levels in newly diagnosed acute myocardial infarction (AMI) may remain high in the survival and the impact of the cardiometabolic risk factors is small.

**Methods:**

Forty patients with AMI are enrolled in this study. The cardiometabolic factors including the anthropometric measurements, blood pressure and lipid profile were determined. The diagnosis of AMI is based on the electrocardiograph, cardiac enzymes and positive troponin-c (cTn) test. Quantitative determination of serum high sensitive C-reactive protein (hs-CRP) and Cys-C was carried out, at the time of admission and at the time of the discharge, using the enzyme-linked immunosorbent assay (ELISA) technique.

**Results:**

Serum Cys-C levels significantly increased at the time of the admission (1,296 ± 431.8 ng/mL) and at the time of the discharge (1,244.6 ± 482 ng/mL) compared with the reference levels (0.7 ± 0.2 ng/mL) of the healthy subjects. Non-significant differences were found between Cys-C levels in respect to the presence or absence of the cardiometabolic risk factors at the times of admission and discharge. Significant decrease of Cys-C levels was found in patients who have negative cTn at the time of discharge compared with corresponding levels at the time of admission.

**Conclusions:**

We conclude that AMI patients have significant high serum levels of Cys-C at the time of admission and the levels significantly decreased in patients with negative cTn test within few days indicating an association between infarct size and the levels of Cys-C.

## Introduction

The cystatins are the reversible competitive inhibitors of C1 cysteine proteases. Cystatin C (Cys-C) is a small protein molecule present in high concentrations in serum, saliva, and seminal, synovial, and cerebrospinal fluids [1]. It is produced and secreted at a constant rate by most nucleated cells and is freely filtered by the glomerular because of its small size [2]. Cys-C is an immumodulator, and stimulates the synthesis of nitric oxide, α-TNF and interleukin-10 [3]. Cys-C is produced and secreted by cardiomyocytes, and its synthesis is elevated when the heart was subjected to ischemia [4]. In patients with acute heart failure, elevated Cys-C above the median level of 1.3 mg/L was associated with significantly higher mortality at 12 months [5]. In one cross-sectional study including 211 asymptomatic metabolic syndrome patients without prior history of coronary heart disease (CAD), the serum levels of Cys-C in patients with asymptomatic CAD were significantly higher than those without CAD, and the levels of serum Cys-C were increased with the increasing of number of disease vessels [6]. Serum uric acid, body mass index and serum triglyceride were independently associated with Cys-C [6]. In patients with ST-elevation myocardial infarction (STEMI), the serum level of Cys-C is increased compared with patients without acute coronary syndrome and it is significantly correlated inversely with ejection fraction in patients with coronary artery disease [7]. One study reported that patients with a history of STEMI and high serum Cys-C levels (> 1,200 ng/mL) who underwent angioplasty were more likely to have increased in-hospital and 1-month cardiovascular mortality [8]. High levels of serum Cys-C (930 - 1,300 ng/mL) were prognostic for death, and for the occurrence of any fatal and non-fatal cardiovascular events for patients with either acute coronary syndrome [9]. Fu et al (2013) reported that a cut-off Cys-C level of 1,605 ng/mL is a considerable predictor of long-term mortality and cardiac events rate in patients with acute coronary syndrome is associated with diabetes mellitus [10]. This study aimed to test the null hypothesis that Cys-C levels in newly diagnosed acute myocardial infarction (AMI) may remain high in the survival and the impact of the cardiometabolic risk factors is small.

## Materials and Methods

This study was conducted in Departments of Medicine, College of Medicine, Diyala University in Iraq. A consent form was obtained from each patient prior to the admission to the study. The study was conducted according to the ethical guidelines constructed by the Scientific Committee of the Institute in which the treatment or using device should not be harmful to the patient and the patient is free to decline from the study or to refuse for study admission.

The patients were recruited from the intensive care unit (ICU) at the General Teaching Hospital in Diyala, where critical patients with cardiac diseases were admitted. Patients with recent AMI were included. The diagnosis of AMI was based on the electrocardiograph (ECG), cardiac enzymes, and positive troponin-c (cTn) test. Exclusion criteria included secondary hypertension, diabetes mellitus, chronic liver diseases, renal disorders, autoimmune diseases and drug intake, e.g. non-steroidal anti-inflammatory drugs.

A total number of 40 patients who presented with chest pain confirmed by cardiac enzymes, ECG findings (ST elevation) and cTn were enrolled in the study. The authors obtained the demographic data, medical history and the treatment data from each patient and recorded the modifiable risk factors, events or complications, and current therapy. A current smoker was defined as a patient who reported smoking on admission. The anthropometric measurements including height (m), weight (kg), waist circumference (cm), and hip circumference were measured. The body mass index (BMI), waist/hip ratio, and waist/height ratio were calculated. The blood pressure (mm Hg) was measured on sitting position and the mean of three readings was taken. The difference between systolic and diastolic blood pressure represented the pulse pressure and the mean arterial blood pressure was equal to diastolic blood pressure + 1/3 pulse pressure.

Peripheral venous blood samples were drawn immediately into tubes on the day of admission, then the blood samples were centrifuged at 2,500 rpm for 10 min, and the sera were separated for determination of fasting lipid profile, and high sensitive C-reactive protein (hs-CRP) and Cys-C.

The determinants of lipid profile included fasting serum total cholesterol (TC), triglycerides (TG), and high density lipoprotein-cholesterol (HDL-c). The low density lipoprotein-cholesterol (LDL-c) is determined by using the equation: TC - (HDL-c + TG/5). The atherogenic index was calculated by estimating the log of the ratio of TG to HDL values.

Another venous blood samples were obtained on the day of hospital discharge, that is, after management of AMI and the patients recovered from critical illness for determination of serum hs-CRP and Cys-C.

Quantitative determination of serum hs-CRP and Cys-C was carried out using the enzyme-linked immunosorbent assay (ELISA) technique. The following hs-CRP values indicated the level of cardiovascular event risk: < 1.0 mg/L (low risk), 1.0 - 3.0 mg/L (intermediate risk) and > 3.0 mg/L (high risk).

Cys-C (human) ELISA kit (DRG Instruments GmbH, Germany) with a detection sensitivity of 0.25 ng/mL is used. The principle of this test is that the Cys-C in the serum sample is reacted with polyclonal anti-human Cys-C antibody in conjugation with horseradish peroxidase enzyme in a microtiter plate wells. The mean ± SD of the reference serum Cys-C levels of healthy subjects (n=30) in our laboratory is 0.7 ± 0.2 ng/mL.

### Statistical analysis

Data are expressed as number, percent, and mean ± SD. Unpaired and paired Student’s *t*-test was used to evaluate differences between the two groups. For all tests, a two-tailed P ≤ 0.05 was considered statistically significant. All calculations were made using Excel 2003 program for Windows.

## Results

A total number of 40 patients (25 men and 15 women) with a mean age of 57.1 years were enrolled in this study. The patients were admitted into the coronary care unit because of chest pain of duration ranged between 2 and 48 h. Table 1 shows the characteristics of patients. cTn test is positive in 25 out of 40 patients and the ECG findings show anterior myocardial infarction with extension to the septal and lateral sites in 75% of patients, while 25% have inferior or inferior-posterior myocardial infarction. Cardiac complications are observed in term of heart failure (40%), and cardiac arrhythmias (35%) and 5% of patients died because of cardiac arrhythmias and cardiogenic shock. In hospital the patients were treated with several therapeutic modalities including thrombolytics (15%), anticoagulants (95%), antianginal (95%) and antiplatelets (100%). Narcotic analgesia was prescribed only to 45% of patients. Table 2 shows the anthropometric measurements in which 42.5% of patients have a BMI of normal built and 57.5% patients are over-weight-obese; the overall mean BMI is 27.2 ± 5.95 kg/m^2^. Seventeen out of 40 patients have the waist circumference of ≥ 102 cm and the means waist/hip, and waist/height ratios are 0.984 and 0.610 respectively. Table 3 shows the blood pressure measurements on the admission in which the means systolic, diastolic and pulse pressures are 139.5, 84.9 and 54.6 mm Hg respectively. Table 4 shows that the mean level of fasting serum triglycerides is 177.4 mg/dL, and 26 out of 40 patients have a serum triglycerides level > 150 mg/dL. Seventeen patients have a low serum high-density lipoprotein levels, eight men (< 35 mg/dL) and nine women (< 50 mg/dL). The mean value of calculated atherogenic index is 0.566.

**Table 1:** Characteristic of Patients Presented With Acute Myocardial Infarction

Characteristics	Number (%), mean ± SD
Gender (M:F)	25:15:00
Age (years)	57.1 ± 8.2
Ethnicity	
Arabs	37 (92.5)
Kurds	2 (5)
Others	1 (2.5)
Race	
White	32 (80)
Black	8 (20)
Onset of chest pain (h)	10.72 ± 9.85 (2 - 48)
Previous history of ischemic heart disease	13 (32.5)
Tropinin C test (positive)	26 (65)
ECG findings	
Anterior	13 (32.5)
Anterolateral	4 (10)
Anteroseptal	13 (32.5)
Inferior	8 (20)
Inferio-posterior	2 (5)
Complications	
Heart failure	16 (40)
Cardiac arrhythmias	14 (35)
Cardiogenic shock	3 (7.5)
Death	2 (5)
Direct current shock	3 (7.5)
Day stay in hospital	3.83 ± 1.38 (2 - 7)
Drug therapy	
Thrombolytics	6 (15)
Anticoagulants	38 (95)
Antianginals	38 (95)
Antiplatelets	40 (100)
Lipid lowering agents	35 (87.5)
Antiarrhythmics	12 (30)
Angiotensin converting enzyme inhibitors	12 (30)
Beta-blockers	22 (55)
Narcotics	18 (45)
Diuretics	14 (35)

The results are expressed as number (%) and mean ± SD.

**Table 2:** Anthropometric Measurements

Variables	Number (%), mean ± SD
Weight (kg)	75.3 ± 16.3
Height (m)	1.665 ± 0.073
Body mass index (kg/m^2^)	27.2 ± 5.95
< 25 kg/m^2^	17 (42.5)
≥ 25 kg/m^2^	23 (57.5)
Waist (cm)	101.45 ± 12.54
≥ 102 cm	17 (42.5)
Hip (cm)	103.1 ± 11.13
Waist/hip ratio	0.984 ± 0.064
Waist/height ratio	0.610 ± 0.082

The results are expressed as number (%) and mean ± SD.

**Table 3:** Blood Pressure Measurements

Blood pressure (mm Hg)	Measurement
Systolic	139.5 ± 18.0
Diastolic	84.9 ± 13.2
Pulse pressure	54.6 ± 10.1
Mean arterial	103.1 ± 14.2

The results are expressed as mean ± SD.

**Table 4:** Fasting Serum Lipid Profiles

Lipid variable	Number (%), mean ± SD
Triglycerides (mg/dL)	177.4 ± 89.5
≥ 150 mg/dL	26 (65)
Cholesterol (mg/dL)	172.9 ± 44.8
High density lipoprotein (mg/dL)	43.7 ± 9.3
< 35 mg/dL (men)	8/25 (32)
< 50 mg/dL (women)	9/15 (60)
Low density lipoprotein (mg/dL)	93.6 ± 47.2
Very low density lipoprotein (mg/dL)	35.5 ± 17.9
Atherogenic index	0.566 ± 0.224

The results are expressed as number (%) and mean ± SD.

On admission, the serum level of hs-CRP was 1.777 ± 0.965 µg/mL and increased non-significantly to 1.944 ± 0.953 µg/mL on discharge from the hospital. Further analysis of the serum hs-CRP level according to the categories of risk on admission showed the patients at low risk (15), intermediate risk (23) and high risk (two). These numbers changed on the hospital discharge to be at low risk (8), intermediate risk (28) and high risk (two). On admission, the mean ± SD serum Cys-C level was 1,296 ± 431.8 ng/mL and decreased non-significantly at the day of hospital discharge, to 1,244.6 ± 482 ng/mL which was significantly higher than the reference levels of the healthy subjects. Further analysis of serum Cys-C level in respect to cardiometabolic risk factors and to cTn marker revealed that the alterations of serum Cys-C level do not relate significantly to cardiometabolic risk factors (Table 5). Patients with positive cTn test have a non-significant high serum Cys-C level compared with negative-cTn patients at the day of admission and a significant decrease of serum Cys-C level at the day of hospital discharge in patients with negative cTn (Table 5).

**Table 5:** Assessment of Serum Cystanin-C Level in Relation to the Cardio-Metabolic Risk Factors and the Cardiac Marker Troponin-C

Cardiometabolic risk factor and cardiac marker	Serum cystatin C level on admission	Serum cystatin C on discharge	P value
Body mass index (kg/m^2^)			
< 25 kg/m^2^	1,191.7 ± 317.3	1,139.3 ± 242.1	0.44
≥ 25 kg/m^2^	1,373.8 ± 492.5	1,322.6 ± 594.9	0.52
Serum triglycerides (mg/dL)			
< 150	1,324.2 ± 360.7	1,166.9 ± 355.7	0.07
≥ 150	1,281.4 ± 471.7	1,286.6 ± 539.6	0.09
Blood pressure			
Systolic (mm Hg)			
≥ 140	1,342.7 ± 499.0	1,303.4 ± 559.1	0.60
< 140	1,210.4 ± 261.4	1,135.6 ± 274.8	0.26
Diastolic (mm Hg)			
≥ 90	1,369.2 ± 536.7	1,348.5 ± 617.7	0.83
< 90	1,223.7 ± 289.0	1,141.0 ± 268.8	0.11
Serum high density lipoprotein (mg/dL)			
≤ 35 (men) or ≤ 50 (women)	1,316.3 ± 545.9	1,272.0 ± 571.8	0.06
> 35 (men) or > 50 (women)	1,283.2 ± 348.3	1,226.5 ± 423.7	0.44
Troponin C			
Positive	1,320.2 ± 448.6	1,369.1 ± 529.1	0.44
Negative	1,263.5 ± 425.5	1,022.1 ± 272.7	0.01*

The results are expressed as mean ± SD. *Significant: compared with corresponding value on the day of hospital admission.

## Discussion

The results of this study show that the serum levels of Cys-C are significantly higher in patients with AMI at the time of the presentation or at the time of the discharge in comparison with the reference levels of healthy subjects. The levels of serum Cys-C show variation in respect to the cardiometabolic risk factors at the onset of AMI. At the time of discharge, the serum levels of Cys-C significantly reduced in patients have a negative cTn. The characteristics of the patients at the admission into the coronary care units may share in the fluctuation of the Cys-C levels. The majority of patients (38 out of 40) have cardiac complications. Yamamoto et al (2013) demonstrated high levels of cystatin patients with cardiovascular events including hospitalization for progressive heart failure [11]. The patients received different modalities of drug therapy that may interfere with the serum levels of Cys-C. Obesity is a cardiometabolic risk factor that links with the levels of Cys-C. Recently, Ito et al (2014) demonstrate that the levels of serum Cys-C can predict the coronary artery disease in obese patients [12]. In this study, more than half of patients have BMI ≥ 25 kg/m^2^ and this explains the significant higher serum levels of Cys-C in comparison with the reference levels of healthy subjects. Despite mean levels of the blood pressure are within the normal range, the serum Cys-C levels are higher than the reference value whether on the admission or on the discharge. Patients with high blood pressure whether systolic or diastolic have non-significant high Cys-C levels than those with normotensive patients. This indicates that the significant high level of Cys-C is associated with AMI (in absence of kidney damage) rather than blood pressure. In patients with renal disease, the high Cys-C level is associated with hypertension [13]. Atherogenic markers in term of high serum triglycerides and low levels of high-density lipoprotein are evident in this study. The non-significant differences between the Cys-C levels at the time of admission and at the time of discharge indicate that the levels of Cys-C relate to the cardiac infarction. This is in agreement with other study that showed a slight increase of Cys-C levels in some patients who presented with dyslipidemia [14]. Patients who have positive cTn test have higher non-significant Cys-C levels compared with negative cTn test at the time of admission. This significant decrease of Cys-C at the time of discharge in patients with negative cTn indicates that there is an association between the Cys-C levels and the infarct size [15]. Recently, De Servi et al reported that Cys-C levels were similar in acute coronary syndrome patients with and without cTn and these levels slightly increased up to the 6 weeks after clinical presentation [16]. The non-significant changes in the serum levels of hs-CRP that are observed in this study are attributed to the short day-stay in hospital which is not sufficient to observe the significant changes.

We conclude that patients with AMI have significant high serum levels of Cys-C at the time of admission and the levels significantly decreased in patients with negative cTn test within few days indicating an association between infarct size and the levels of Cys-C.
